# Prevalence and temporal trends in prepregnancy nutritional status and gestational weight gain of adult women followed in the Brazilian Food and Nutrition Surveillance System from 2008 to 2018

**DOI:** 10.1111/mcn.13240

**Published:** 2021-07-13

**Authors:** Thaís Rangel Bousquet Carrilho, Kathleen M. Rasmussen, Jennifer A. Hutcheon, Ronaldo Fernandes Santos Alves, Dayana Rodrigues Farias, Nathalia Cristina Freitas‐Costa, Mylena Maciel Gonzalez, Mônica Araújo Batalha, Gilberto Kac

**Affiliations:** ^1^ Nutritional Epidemiology Observatory, Josué de Castro Nutrition Institute Federal University of Rio de Janeiro Rio de Janeiro Brazil; ^2^ Division of Nutritional Sciences Cornell University Ithaca New York USA; ^3^ Department of Obstetrics and Gynaecology, Faculty of Medicine The University of British Columbia Vancouver British Columbia Canada

**Keywords:** anthropometry, body mass index, gestational weight gain, maternal health, pregnancy, public health surveillance, weight gain

## Abstract

Prepregnancy body mass index (BMI) and gestational weight gain (GWG) are the most investigated indicators of maternal nutritional status, which is a modifiable factor that plays a vital role in maternal and infant health. This study describes prepregnancy BMI and GWG of 840,243 women with 2,087,765 weight observations in the Brazilian Food and Nutrition Surveillance System from 2008 to 2018. Prepregnancy BMI was classified according to the World Health Organization cut‐offs. Total GWG was calculated from weight measurements taken after 36 weeks of pregnancy and classified according to the Institute of Medicine guidelines. Temporal trends in prepregnancy BMI status were examined, and maps were used to evaluate changes in excessive GWG in each Brazilian federation unit. On overall, prepregnancy overweight and obesity increased from 22.6% to 28.8% and from 9.8% to 19.8%, respectively, between 2008 and 2018. The prevalence of excessive GWG rose from 34.2% to 38.7% during the same period and in 11 of the 27 Brazilian federation units between 2008 and 2016. Women with underweight showed the highest values for mean total GWG for all the compared years (overall variation from 12.3 to 13.1 kg), followed by those with normal weight (11.9 to 12.5 kg), overweight (10.1 to 10.9 kg) and obesity (from 8.2 to 8.9 kg). Within each BMI group, values remained fairly stable throughout the studied period for first‐ and second‐trimester GWG and total GWG. These results help to fill a significant gap in understanding the distribution of prepregnancy BMI and GWG in Brazilian women.

**Key messages**

- In data from the national surveillance system in Brazil, prepregnancy BMI increased substantially from 2008 to 2018. Overweight increased from 22.6% to 28.8%, whereas obesity doubled from 9.8% to 19.8%.
- The prevalence of GWG within the IOM guidelines was fairly stable throughout the studied period at approximately 33%. Still, the mean total GWG presented a slight increase (~1 kg) between 2008 and 2018 for all BMI categories.
- The prevalence of GWG above the IOM guidelines increased for 11 of the 27 Brazilian federation units.

## 1 INTRODUCTION

Prepregnancy weight status is an important consideration during prenatal care because of its association with maternal and child adverse health outcomes (Aune et al., 2014; Yu et al., 2013). The prevalence of overweight or obesity according to body mass index (BMI) has increased among women of reproductive age in the last 30 years, especially in high‐income countries (HIC) (Popkin & Slining, 2013; Poston et al., 2016). This is also true for low‐ and middle‐income countries (LMIC), such as Brazil, where the prevalence of excessive weight (overweight and obesity) for non‐pregnant adult women is estimated to be over 50% in several states (Ministério da Saúde, 2020). However, national‐level data on temporal changes in prepregnancy BMI are scarce.

One of the main manifestations of pregnancy is an increase in maternal weight. Gestational weight gain (GWG) is normal and reflects both adequate growth and development of the fetus and the changes in women's bodies to support pregnancy and subsequent lactation (Institute of Medicine [IOM] [US] and National Research Council [US] Committee to Reexamine IOM Pregnancy Weight Guidelines, 2009). Health professionals often monitor GWG during prenatal care because excessive and inadequate weight gain is linked with adverse outcomes (Goldstein et al., 2017, 2018; Kominiarek & Peaceman, 2017).

The most commonly used guidelines for GWG are those established by the IOM in the United States (IOM [US] and National Research Council [US] Committee to Reexamine IOM Pregnancy Weight Guidelines, 2009). These guidelines were incorporated by the Brazilian Ministry of Health to be used during prenatal care since 2011 (Ministério da Saúde, 2011). Studies that show the prevalence of GWG below IOM guidelines for LMIC are scarce. The few available studies show that GWG below these guidelines is common, which indicates that low GWG is a serious problem for many LMIC (Asefa & Nemomsa, 2016; Esimai & Ojofeitimi, 2014; Gondwe et al., 2018; Young et al., 2017). In contrast, the prevalence of GWG above these guidelines is higher in HIC (Goldstein et al., 2018; Kominiarek & Peaceman, 2017).

Since 2008, the Brazilian population's nutritional status has been monitored using the National Food and Nutrition Surveillance System (SISVAN) (Ministério da Saúde, 2013). The system was designed to record population‐level information on nutritional status and food consumption, including pregnant women who used public prenatal care services (Ministério da Saúde, 2011). The data collected are used as a basis for the development and evaluation of public health nutrition policies for the country.

To the best of our knowledge, there are no studies with population‐based data that describe prepregnancy BMI and GWG per trimester and their changes over time in LMIC. Monitoring the trends in those indicators is essential to formulate and redefine policies to reduce and prevent adverse outcomes associated with the deviation in the maternal nutritional status before and during pregnancy. Thus, we aimed to describe the prevalence and temporal trends in prepregnancy BMI and GWG in adult pregnant women followed in the SISVAN from 2008 to 2018.

## 2 METHODS

### 2.1 Data and main variables

We used data from the SISVAN, an administrative system run by the Brazilian Ministry of Health that collects anthropometric and sociodemographic data in all the human life cycle phases, from 2008 to 2018 (not the entire year). For pregnant women, the data are collected during routine prenatal care. Measurements follow a standardized protocol (Ministério da Saúde, 2011) and are commonly collected by health care professionals who work in primary care settings.

The sociodemographic and prenatal care variables used in this study were maternal age, maternal education, participation in the Bolsa Familia programme (PBF), gestational age in the prenatal visits and the number of prenatal care visits. Maternal age was calculated as the difference between the date of the LMP and the date of women's birth. Maternal education (years of schooling) was collected from the system form and categorized as ≤1, 1–9, 10–12 and >12 years (OECD/Eurostat/UNESCO Institute for Statistics, 2015). Participation in the PBF, which is the national conditional cash transfer implemented in Brazil since 2004 (Ministério da Saúde, 2013), was classified as yes/no. Gestational age (weeks) in the prenatal visit was calculated as the difference between the date of the visit and the LMP date, which is the only information available to calculate gestational age. The number of prenatal care visits for each woman was determined as the total of visits in the same pregnancy.

Prepregnancy weight was self‐reported and, together with the first measurement of height (in metres) taken during pregnancy, was used to calculate prepregnancy BMI (kg/m^2^). The World Health Organization (WHO) cut‐offs were used for BMI classification as underweight (<18.5 kg/m^2^), normal weight (18.5–24.9 kg/m^2^), overweight (25.0–29.9 kg/m^2^) and obesity (≥30.0 kg/m^2^) (WHO, 1995).

GWG was calculated as the difference between weight measured in each visit and self‐reported prepregnancy weight. Whenever there was more than one weight measurement in a trimester for a given woman, the last measurement taken within the trimester was used. Total GWG was classified as ‘below’, ‘within’ or ‘above’ the guidelines, according to the values proposed by the IOM (US) and National Research Council (US) Committee to Reexamine IOM Pregnancy Weight Guidelines (2009). Only measurements taken after 36 weeks of pregnancy were used for calculating this variable. The classification of total GWG considered the expected GWG at the exact gestational age of the measurement and not at 40 weeks (which is the value used in the guidelines). As an example, the upper limit of the recommendations for a normal‐weight woman who had her last weight measured at the 38th week should be calculated as 2 kg for the first trimester (13 weeks) + 0.5 kg/week (the IOM rate for normal‐weight women) × 25 weeks (38–13 weeks, the gestational age difference between the weight measurement and the end of the first trimester) = 14.5 kg. This adjustment in the IOM ranges aims to consider the gestational age at the measurement and is essential because the misclassification of GWG resulting from varying lengths of gestation is possible (Hutcheon et al., 2012).

For variables that remained constant during pregnancy (e.g., maternal age, education, prepregnancy BMI and participation in the PBF), only information from the first visit in each pregnancy was used.

### 2.2 Data cleaning process

Because the system is based on data collected in routine prenatal care, several steps to clean the data were implemented. To improve data quality, we removed duplicates and identified implausible maternal dates of birth, dates of last menstrual period (LMP) and dates of clinical appointments based on chronological plausibility. We checked and reorganized the data for categorical variables, such as maternal schooling. For instance, there were several different codes used for missing data (such as ‘NOT INFORMED’, ‘MISSING’ and ‘NA’), and the codes for these variables were standardized. After recodifying the missing data, in variables considered stable throughout pregnancy, such as maternal education, age and participation in the PBF, we used single‐imputation techniques to complete each woman's data. Next observation carried backward (NOCB) and last observation carried forward (LOCF) were used to complete missing data on specific visits, whenever the data were available for that woman in any of the pregnancy visits. For example, if a woman presented data on education in the first visit and not in the subsequent ones, we used LOCF to complete the data from the other visits because we considered it to be unlikely that she would have changed her educational status during the same pregnancy. The implementation of those single‐imputation techniques was possible because a portion of the dataset presented repeated measurements.

We implemented two methods to identify outliers for weight at each clinical appointment. These steps considered the plausibility of the measurement relative to previous and subsequent weight measurements. Specifically, we employed the conditional approach method proposed by Yang and Hutcheon (2016) and the jackknife residuals method proposed by Shi et al. (2018). We identified implausible values in cross‐sectional data by classifying the measurements using an external reference chart and identified outliers based on extreme z‐scores. Height was classified using the WHO height‐for‐age charts for 19‐year‐old women (de Onis et al., 2007), and z‐scores >6 or <−6 were flagged as implausible. For prepregnancy BMI, WHO BMI‐for‐age charts (de Onis et al., 2007) were used and z‐scores >5 or <−5 were flagged. For GWG, the INTERGROWTH‐21st GWG chart for normal‐weight women (Cheikh‐Ismail et al., 2016) was used, and for all BMI categories, z‐scores >6 or <−6 from that chart were considered outliers. Women with GWG above or below 4 standard deviations (SDs) of the internal sample distribution according to each trimester were also flagged as outliers. To decide which values of z‐scores would be flagged and removed for each variable, the plausibility of the distribution in the remaining data and the proportion of data to be removed were considered.

### 2.3 Statistical analyses

Continuous variables were summarized using mean and SDs and categorical variables with absolute and relative frequencies. Maps to evaluate changes in the prevalence of excessive GWG in each Brazilian state in triennials (2008–2010, 2011–2013 and 2014–2016) were created. In these maps, only states with *n* > 100 women in each triennial were included. We did not perform any statistical tests to compare the variation in the key variables throughout the years because the large sample size would mean that even small differences would be statistically significant while not being clinically important.

The analyses were performed in JupyterHub from ‘Plataforma de Ciência de Dados aplicada à Saúde’ (ICICT/Fiocruz), using r Version 3.6. For the creation of the maps, r package ‘brazilmaps’ was used.

### 2.4 Ethical considerations

The Research Ethics Committee of the Rio de Janeiro Federal University Maternity Teaching Hospital approved the project from which this study is part (protocol number: 85914318.2.0000.5275). All analyses were conducted with deidentified data, and security procedures to protect the access to the data were taken.

## 3 RESULTS

The initial dataset contained 5,178,974 different participants and 22,291,787 weight records. We removed the 10,613,874 duplicate records that resulted from combining data from three sources (i.e., the SISVAN data, data from e‐SUS and the PBF database) and 603 records from 2007 that appeared in the registers, which left 11,677,310 records. After identifying and removing outliers and selecting women with prepregnancy weight information, there were 840,243 women with 2,087,765 weight records for analysis (Figure S1). The high proportion of women removed in the final step (approximately 84% of the initial dataset) primarily resulted from missing data in the prepregnancy weight field, which is not mandatory in the SISVAN form and was often left blank.

Maternal mean age in the first registered visit increased slightly from 2008 to 2018 (26.3 to 27.0 years, respectively). The frequency of women with 10 to 12 or >12 years of education registered in the system increased substantially during the evaluated period (<1% in 2008 to approximately 50% in 2018). Also, the percentage of women who were part of the PBF increased from 38.4% in 2008 to 43.7% in 2018. The mean number of visits registered in the system increased from 3 to 4 during this period (Table 1). This increase was also observed for the median of this variable, which varied from 2 (interquartile range [IQR] 1–4) in 2008 to 3 (IQR 2–6) in 2018 (data not shown).

**Table 1:** Sociodemographic, prenatal care and anthropometric characteristics of Brazilian pregnant women registered in the Food and Nutrition Surveillance System (SISVAN) between 2008 and 2018

Continuous variables	Mean (SD)										
	2008	2009	2010	2011	2012	2013	2014	2015	2016	2017	2018^a^
Number of records	69,413	157,680	178,033	232,617	266,640	250,386	218,177	230,838	219,411	193,962	70,614
Sociodemographic											
Maternal age at first visit (years)	26.3 (5.9)	26.4 (5.8)	26.5 (5.9)	26.5 (5.8)	26.6 (5.9)	26.6 (5.9)	26.7 (5.9)	26.8 (6.0)	26.8 (6.0)	27.0 (6.0)	27.0 (6.0)
Prenatal care											
Number of visits (with weight measurement)	3.0 (5.4)	3.6 (5.5)	3.8 (5.5)	4.1 (5.5)	4.0 (5.5)	4.0 (5.4)	4.1 (5.3)	4.4 (5.4)	4.6 (5.3)	4.5 (5.4)	4.0 (5.4)
Gestational age in the first registered visit	20.4 (9.8)	18.8 (9.5)	17.3 (9.1)	16.8 (9.1)	16.5 (9.1)	16.5 (9.1)	16.3 (9.1)	16.3 (9.1)	15.4 (8.8)	15.6 (9.0)	15.4 (8.9)
Anthropometric											
Self‐reported prepregnancy weight (kg)	59.9 (12.1)	60.8 (12.5)	61.5 (12.8)	62.3 (13.1)	62.9 (13.3)	63.1 (13.3)	63.8 (13.6)	64.6 (13.9)	65.1 (14.1)	65.6 (14.3)	65.9 (14.6)
Height (cm)	158.4 (6.5)	158.9 (6.5)	159.1 (6.5)	158.2 (6.5)	159.3 (6.5)	159.4 (6.4)	159.6 (6.4)	159.9 (6.4)	160.0 (6.4)	160.0 (6.4)	160.0 (6.4)
Prepregnancy BMI (kg/m^2^)	23.9 (4.5)	24.1 (4.6)	24.3 (4.7)	24.5 (4.8)	24.7 (4.9)	24.8 (4.9)	25.0 (5.0)	25.2 (5.1)	25.4 (5.2)	25.6 (5.3)	25.7 (5.4)
Gestational weight (kg)	64.5 (12.7)	64.8 (13.0)	65.0 (13.2)	65.6 (13.5)	66.1 (13.7)	66.1 (13.6)	66.8 (13.9)	67.5 (14.2)	67.6 (14.2)	68.2 (14.6)	68.6 (14.8)

Categorical variables	*n* (%)										
	2008	2009	2010	2011	2012	2013	2014	2015	2016	2017	2018^a^
Maternal education (years)											
≤1	8105 (37.3)	15,282 (34.7)	15,313 (32.5)	16,631 (29.3)	17,158 (27.9)	14,254 (25.5)	10,991 (24.5)	13,053 (22.0)	6524 (15.5)	2356 (8.3)	689 (6.3)
1–9	13,501 (62.2)	28,505 (64.7)	31,496 (66.8)	39,670 (69.9)	43,773 (71.1)	40,827 (73.1)	32,919 (73.4)	29,034 (48.9)	21,036 (50.1)	13,429 (47.5)	4774 (44.0)
10–12	99 (0.5)	246 (0.6)	266 (0.6)	395 (0.7)	532 (0.9)	597 (1.1)	763 (1.7)	14,338 (24.2)	12,022 (28.6)	10,188 (36.1)	4398 (40.5)
>12	7 (0.03)	32 (0.07)	56 (0.11)	69 (0.1)	100 (0.1)	147 (0.3)	188 (0.4)	2892 (4.9)	2424 (5.8)	2299 (8.1)	998 (9.2)
Participation in ‘Bolsa Familia’											
Yes	15,702 (38.4)	25,908 (34.0)	27,244 (33.6)	33,927 (33.7)	40,866 (35.3)	34,410 (31.4)	37,988 (42.3)	36,839 (42.2)	33,385 (43.3)	31,038 (45.3)	10,930 (43.7)
No	25,223 (61.6)	50,217 (66.0)	53,800 (66.4)	66,657 (66.3)	74,940 (64.7)	75,359 (68.6)	51,797 (57.7)	50,388 (57.8)	43,711 (56.7)	37,491 (54.7)	14,062 (56.3)

^a^ Data refer to the incomplete year.

At the first antenatal care registered visit, the median gestational age decreased substantially from nearly 20 weeks in 2008 to <13 weeks in 2018. In 2008, the top quartile of the sample distribution had their first antenatal visit at or after 28 gestational weeks, a value that dropped to approximately 21 gestational weeks in 2018 (Figure S2). However, only 48% of the women presented ≤12 weeks in the first registered visit, and half of the women had only one visit registered in the system.

**Figure 1:**
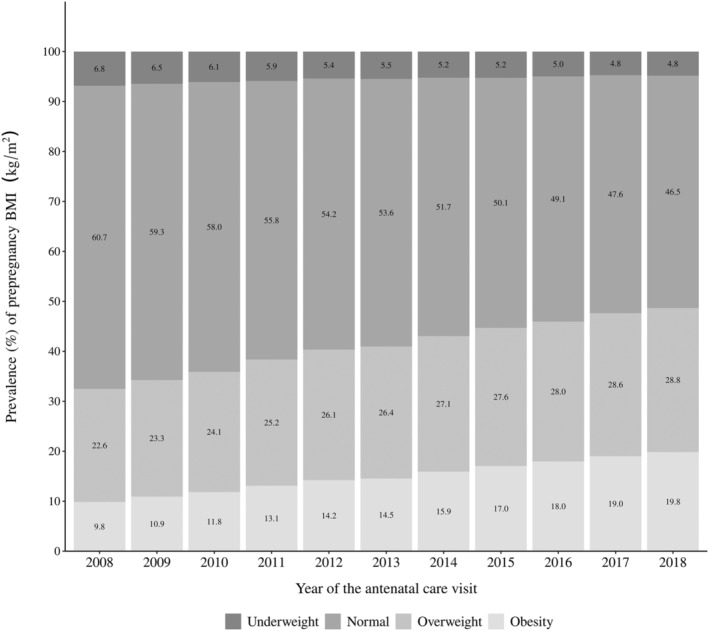
Prevalence of prepregnancy body mass index (BMI) of Brazilian women followed in the Food and Nutrition Surveillance System (SISVAN), 2008–2018. BMI cut‐offs: underweight, BMI < 18.5 kg/m^2^; normal, BMI ≥ 18.5 and <25.0 kg/m^2^; overweight, BMI ≥ 25.0 and <30.0 kg/m^2^; and obesity, BMI ≥ 30.0 kg/m^2^

Prepregnancy BMI increased substantially throughout the studied period. Overweight increased from 22.6% to 28.8% and obesity from 9.8% to 19.8%, respectively, from 2008 to 2018. These results show a 21% and 202% increase in the prevalence of overweight and obesity, respectively, in the period. In contrast, the prevalence of women classified as normal weight decreased from 60.7% to 46.5%, whereas the prevalence of underweight decreased from 6.8% to 4.8% (Figure 1).

**Figure 2:**
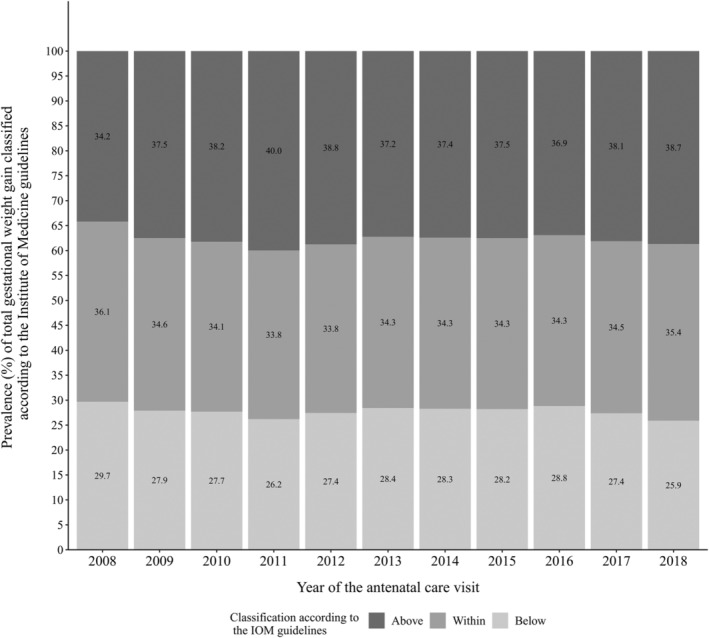
Prevalence of total gestational weight gain (GWG) classified according to the Institute of Medicine (IOM) guidelines, Food and Nutrition Surveillance System (SISVAN), 2008–2018. GWG was calculated as the difference between the weight measured in the prenatal visit and self‐reported prepregnancy weight. Only measurements taken after 36 weeks were considered

According to the IOM, approximately one third of the participants had GWG classified as ‘within guidelines’, and this proportion was fairly stable throughout the studied period. There was a slight increase in the prevalence of GWG above the guidelines from 34.2% to 38.7%, which coincided with a reduction of women gaining weight below the guidelines from 29.7% to 25.9%, from 2008 to 2018, respectively (Figure 2).

**Figure 3:**
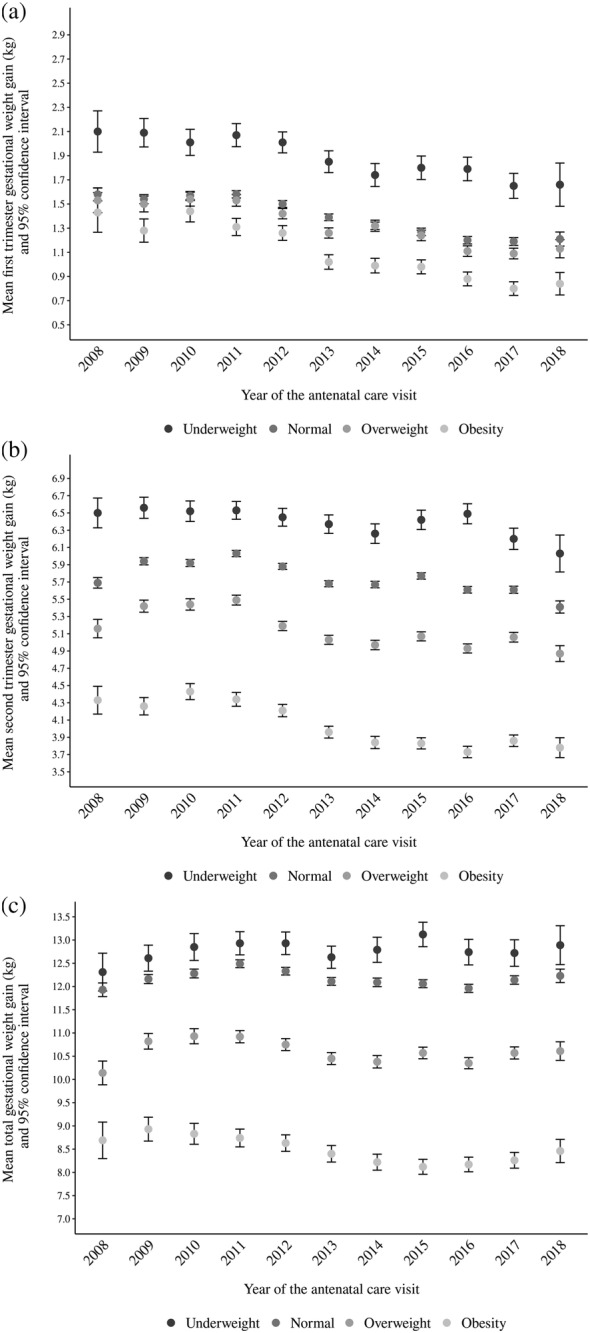
Mean gestational weight gain (GWG) and 95% confidence intervals according to prepregnancy body mass index (BMI) category, Food and Nutrition Surveillance System (SISVAN), 2008–2018: (a) first‐trimester GWG, (b) second‐trimester GWG and (c) total GWG. BMI cut‐offs: underweight, BMI < 18.5 kg/m^2^; normal, BMI ≥ 18.5 and <25.0 kg/m^2^; overweight, BMI ≥ 25.0 and <30.0 kg/m^2^; and obesity, BMI ≥ 30.0 kg/m^2^. GWG was calculated as the difference between the weight measured in the prenatal visit and self‐reported prepregnancy weight. For total GWG, only measurements taken after 36 weeks were considered

Mean total GWG varied according to prepregnancy BMI. Women with underweight women showed the highest values (variation from 12.3 to 13.1 kg between 2008 and 2018), followed by normal weight (variation from 11.9 to 12.5 kg), overweight (variation from 10.1 to 10.9 kg) and obesity (variation from 8.2 to 8.9 kg). Within each BMI category, the variation was below 1 kg throughout the studied period. The pattern for the first and second trimesters was similar to total GWG (Figure 3).

**Figure 4:**
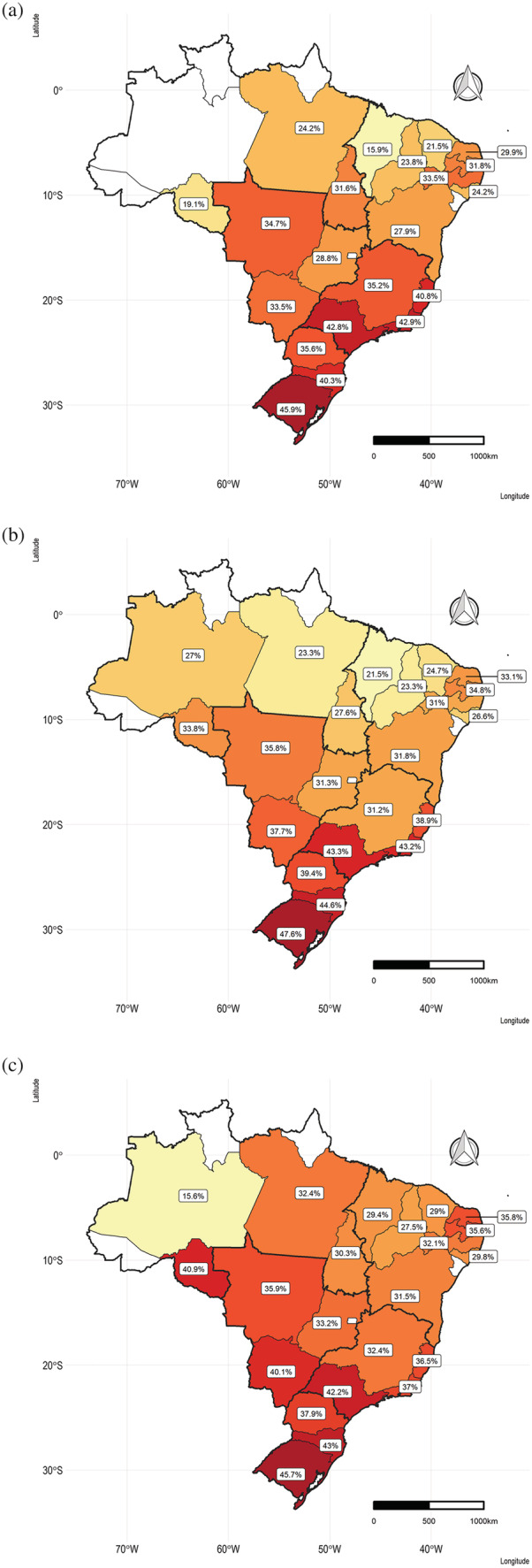
Prevalence of gestational weight gain above the Institute of Medicine guidelines per Brazilian state, Food and Nutrition Surveillance System (SISVAN), 2008–2016: (a) 2008‐2010. (b) 2011‐2013. (c) 2014‐2016. Only states with at least 100 observations in all periods were included

Excessive GWG became more common in the vast majority of the Brazilian states throughout the study period. Brazil's South Region was the only region with a prevalence of excessive GWG above 45% since the first triennial (2008–2010), a situation that has remained the same throughout the studied period. The Southeast Region had the second highest prevalence of excessive GWG, even though a reduction in the most recent triennial was observed in three of the four states of the region. Excessive GWG increased for almost all states in the Northeast and Center‐West regions (Figure 4).

## 4 DISCUSSION

Using routine prenatal care data available in Brazil's SISVAN public health database, we observed a 21% increase in the prevalence of prepregnancy overweight and a 202% increase in prepregnancy obesity between 2008 and 2018. GWG above the IOM guidelines increased 11.6% throughout the years in this sample of Brazilian women. We also found a slight increase in the mean total GWG in all BMI categories during the study period. The use of this national surveillance system represents a unique opportunity to investigate the trends in the anthropometric profile of pregnant women in Brazil, where only limited information has been available before and during pregnancy. This is also true for other LMIC, where information on the distribution of anthropometric status throughout gestation and over time is not commonly available.

The trends in the prevalence of prepregnancy BMI of overweight and obesity observed in the current study are similar to those in Brazilian adult women in general, namely, an increase in the prevalence of overweight and obesity in the last 40 years (Instituto Brasileiro de Geografia e Estatística—IBGE, Diretoria de Pesquisas, & Coordenação de Trabalho e Rendimento, 2010). Data from the Risk and Protective Factors Surveillance System for Chronic Noncommunicable Diseases Through Telephone Interview (VIGITEL) 2019 show that the prevalence of excessive weight (BMI > 25 kg/m^2^) in adult women varied between 44% and 61% in the Brazilian capitals, which is similar to our findings for 2018 (48.6%) (Ministério da Saúde, 2020). This trend is also consistent with that observed in women from other LMIC, especially those from Latin America, the Caribbean, the Middle East and North Africa (Popkin & Slining, 2013), where the increase in the rates of obesity seems to be faster than the observed in HIC (Ford et al., 2017; Poston et al., 2016).

The mean total GWG observed in Brazil in 2018 (11.1 kg for all women) is similar to values observed in Mexico (11.7 kg) (Zonana‐Nacach et al., 2010) and Uganda (10.6 kg) (Wanyama et al., 2018) but lower to that reported from China in 2016 (15.6 kg) (Xiao et al., 2017). In a recent study, Carrilho, Farias, et al. (2020) used pooled data from Brazilian studies conducted between 1990 and 2016 and found a mean total GWG of 12.7 kg, which is also close to the mean total GWG observed in the current study. However, the slight reduction in the mean first‐ and second‐trimester GWG observed in the SISVAN data is a unique feature of the Brazilian population and has not been reported elsewhere. Although there was a reduction in the mean GWG in the first and second trimesters for all BMI categories in the current study, this was not translated into a decrease in the mean total GWG. We observed an increase of approximately 1 kg in the mean total GWG from 2008 to 2018 for all BMI categories. These results indicate that most of the studied women are gaining (not losing) weight, which is true even for those classified with obesity before pregnancy.

Although the prevalence of GWG outside of the guidelines was stable in the period, the proportion of women gaining weight above the recommended ranges was high (varying from 34% in 2008 to 38% in 2018). The prevalence of GWG above the guidelines was more pronounced in several states and was above 40% in some regions. GWG above the IOM guidelines has been linked to post‐partum weight retention and maternal obesity (Nehring et al., 2011). This becomes more relevant because of the increase in the prevalence of overweight and obesity observed among women, which may be partially connected to the high prevalence of women who exceeded the weight gain guidelines during pregnancy. On the other hand, the prevalence of women who gained below the guidelines was also high, that is, a quarter of the assessed women in 2018. This proportion is consistent with the prevalence of GWG below the guidelines observed in HIC, such as the Republic of Korea (21%), the United States (21%) and Canada (17%) (Kominiarek & Peaceman, 2017; Subhan et al., 2019; Wie et al., 2017). In LMIC such as Nigeria, Malawi, Vietnam and Ethiopia, the prevalence of women gaining weight below the guidelines is remarkably superior to the values observed in this study (≥70% for the four countries) (Asefa & Nemomsa, 2016; Esimai & Ojofeitimi, 2014; Gondwe et al., 2018; Young et al., 2017). These results show that Brazil seems to be following the trends of increase in the GWG above the guidelines observed in HIC. Ensuring that women gain within the guidelines can help prevent maternal and infant adverse outcomes. The continuous monitoring of this indicator could help defining strategies and programmes aiming to improve that scenario.

The increase in the frequency of participants from the PBF registered in the system is consistent with data from the Ministry of Social Development, which show a general increase in the number of families who are beneficiaries of the programme since 2003, mainly due to an expansion on the programme (ENAP, 2018). The changes observed in maternal education are also similar to the trends observed in the general Brazilian population in the last decades, corroborating the data from the VIGITEL from 2006 to 2016 that also revealed an increase in the frequency of the population with primary education (1–9 years) or more (Cruz et al., 2018). The increase in the percentage of women who are part of the PBF could have helped in the variation observed in prepregnancy BMI and GWG. This may be because recipients of conditional cash transfer programmes seem to be more vulnerable to the nutritional transition and more affected by the epidemic of chronic diseases (Fernald et al., 2008; Martins et al., 2013).

The mean number of prenatal visits increased from 3 to 4. However, this value is still below 6, the number recommended by the Ministry of Health and the WHO (Ministério da Saúde, 2012). This finding reinforces the need to increase the coverage of the SISVAN because recent studies show that the majority of Brazilian women attend six or more prenatal visits during pregnancy (Tomasi et al., 2017; Viellas et al., 2014). This poses a significant limitation when generalizing the study findings because not all the prenatal visits were registered in the system, and it is not possible to identify the registration selection criteria; that is, it is not possible to know how the health care professional decides when or not to include the information of the visit in the system.

The observed decrease in the gestational age in the first registered visit is an important indicator of the adequacy of prenatal care. In ‘Birth in Brazil’, a nationwide study conducted in 2011–2012 that aimed to investigate the characteristics of births in the country, Viellas et al. (2014) observed that only 60.6% of women started prenatal care during the first trimester. Although the median gestational age of the first visit (from 19.1 weeks in 2008 to 12.6 weeks in 2018) was observed in the current study, only 48% of the women presented for their prenatal visit at ≤12 weeks. However, it is not possible to identify whether there has been a real change in those indicators or if this change was only an improvement in the quality of the system.

### 4.1 Strengths and limitations

The large sample size, the availability of data for 10 years and repeated weight measurements for the women are important features of this study. The methods used to identify outliers and to clean the dataset, although responsible for the removal of a large number of women and records, ensured that the analyses were conducted with more reliable data. Moreover, these findings represent an important source of information for other LMIC with similar characteristics to the population included in the SISVAN because studies on the trends of GWG and prepregnancy BMI are limited in those countries.

There are some limitations worth mentioning, however. First, the coverage of the SISVAN is low, considering that there are approximately 2.9 million births each year in Brazil (Instituto Brasileiro de Geografia e Estatística [IBGE], 2018). This is an important limitation for generalizing the findings of this study to the whole population. As a result of the lack of data in Brazil, it is not possible to know the extent to which these data reflect trends in the national obstetrical population. However, the SISVAN data may represent an important part of the population, namely, the beneficiaries of conditional cash transfer programmes and users of the public health care system, for whom the public health care policies are developed and to whom they are directed.

Some initiatives have been developed recently to increase the coverage of the system (Nascimento et al., 2017), but it is still far from ideal. Unfortunately, because the system is based on routinely collected data on prenatal care, the protocol that recommends standardization for anthropometric data collection is not always followed. This results in a lack of high‐quality measurements, which could be both imprecise and inaccurate. To address these challenges to the reliability of the data, we implemented several methods to identify extreme and implausible values before analysing the anthropometric data. These procedures ensured that we worked with more reliable data with plausible distributions.

The form used to collect the data in routine prenatal care reported in the SISVAN includes some non‐mandatory fields, such as prepregnancy weight. This led to a high proportion of missing data in the variables based on those fields. In the future, it is important that this type of information should become mandatory and that the system to enter the information should not accept implausible options, such as ‘9999’ or zero, to reduce the proportion of missing data in key variables.

Finally, we calculated both prepregnancy BMI and GWG based on self‐reported prepregnancy weight, and bias could have been introduced due to this measurement. However, the results from a previous study conducted by our team in a subsample of the same SISVAN data revealed that self‐reported prepregnancy weight has a good agreement with the weight measured in the first trimester, especially when the latter was performed up to 6 weeks of pregnancy (Carrilho, Rasmussen, et al., 2020).

## 5 CONCLUSION

The trends observed in the prevalence of prepregnancy BMI and GWG outside of the IOM guidelines indicate that the nutritional status of these women has worsened in recent years. These findings reveal the need for continuous monitoring of these indicators and the urgent development of strategies to reverse the observed trends. The changes in sociodemographic and prenatal care characteristics observed in the present study suggest an improvement in maternal education, participation in a conditional cash transfer programme and access to prenatal care in early pregnancy.

The use of administrative data for research purposes, although challenging, represents a unique opportunity to evaluate trends in the prevalence of important indicators among pregnant women, such as BMI and GWG. Thus, effective strategies to improve the quality of the data, such as full completion of the data forms, should be made by the Ministry of Health so that the SISVAN data can be used with confidence to generate evidence‐based policies for the country. Finally, the quantity of data and the ability to examine trends over time help to fill an important gap in understanding the distribution of prepregnancy BMI and GWG in women who live in LMIC.

## CONFLICTS OF INTEREST

The authors declare that they have no conflicts of interest.

## CONTRIBUTIONS

TRBC analysed and interpreted the data and wrote the manuscript, with input from all authors. KMR and JAH contributed to the interpretation of the data and revision of the manuscript. RFSA, DRF, NCFC, MMG and MAB contributed to the data cleaning phase, to the discussion of the results and to the revision of the manuscript. GK coordinated the study and participated in all phases of analysis and interpretation of the data and writing of the manuscript. All authors read and approved the final manuscript.

## Supporting information

Supplementary table 1. Distribution of sample size (number of records) by Brazilian state (total) and year registered in the Food and Nutrition Surveillance System (SISVAN), 2008 – 2018.

**Figure S1.** Flowchart for the cleaning steps and constitution of the dataset used in the analyses.

**Figure S2.** Median and interquartile ranges for gestational age in the first prenatal visit in the Food and Nutrition Surveillance System (SISVAN), 2008–2018.

## Data Availability

The data that support the findings of this study are available from the Brazilian Ministry of Health. Restrictions apply to the availability of these data, which were used under licence for this study. Data are available upon request to the Ministry of Health, following specific Brazilian laws.
