# Supplementary material for: Prevalence and temporal trends in prepregnancy nutritional status and gestational weight gain of adult women followed in the Brazilian Food and Nutrition Surveillance System from 2008 to 2018
Source: Matern Child Nutr. 2021 Jul 13;18(1):e13240. doi: 10.1111/mcn.13240 (PMC8710119; doi:10.1111/mcn.13240)
Supplement: Supplementary file 1 — Supplementary table 1. Distribution of sample size (number of records) by Brazilian state (total) and year registered in the Food and Nutrition Surveillance System (SISVAN), 2008 – 2018. [file MCN-18-e13240-s003.docx]

**Prevalence and temporal trends in pre-pregnancy nutritional status and gestational weight gain of adult women followed in the Brazilian Food and Nutrition Surveillance System (SISVAN) from 2008 – 2018**

**SUPPLEMENTARY APPENDIX**

Supplementary table 1. Distribution of sample size (number of records) by Brazilian state (total) and year registered in the Food and Nutrition Surveillance System (SISVAN), 2008 - 2018.

| **State** | **Total** | **2008** | **2009** | **2010** | **2011** | **2012** | **2013** | **2014** | **2015** | **2016** | **2017** | **2018** |
| --- | --- | --- | --- | --- | --- | --- | --- | --- | --- | --- | --- | --- |
| AC | 515 | 4 | 31 | 5 | 47 | 48 | 43 | 52 | 62 | 163 | 60 | 0 |
| AL | 37963 | 611 | 1181 | 4208 | 5426 | 5983 | 5523 | 3467 | 3145 | 2905 | 3760 | 1754 |
| AM | 3673 | 286 | 120 | 204 | 677 | 335 | 422 | 391 | 373 | 420 | 325 | 120 |
| AP | 1180 | 3 | 29 | 20 | 23 | 87 | 89 | 455 | 144 | 144 | 18 | 168 |
| BA | 115979 | 6085 | 8619 | 13078 | 15946 | 19302 | 16370 | 11242 | 9452 | 7933 | 5753 | 2199 |
| CE | 21407 | 1503 | 2079 | 1252 | 1981 | 3887 | 2152 | 1199 | 1422 | 1605 | 2741 | 1586 |
| DF | 2166 | 62 | 154 | 69 | 149 | 284 | 270 | 219 | 287 | 486 | 135 | 51 |
| ES | 61054 | 4045 | 7400 | 5839 | 7911 | 10488 | 7866 | 4352 | 4700 | 4741 | 2521 | 1191 |
| GO | 11742 | 546 | 2188 | 1238 | 1373 | 2053 | 1443 | 1170 | 819 | 427 | 408 | 77 |
| MA | 4964 | 256 | 565 | 220 | 424 | 1110 | 802 | 257 | 585 | 414 | 219 | 112 |
| MG | 421789 | 2812 | 12021 | 13640 | 16973 | 33991 | 53690 | 65297 | 75005 | 68392 | 59948 | 20020 |
| MS | 36006 | 1497 | 3638 | 2255 | 4133 | 5011 | 3832 | 2953 | 2960 | 3918 | 4079 | 1730 |
| MT | 29556 | 1677 | 2920 | 2549 | 4428 | 5051 | 3609 | 2264 | 2439 | 2420 | 1919 | 280 |
| PA | 43620 | 2103 | 5012 | 3449 | 2996 | 3896 | 4872 | 3933 | 4915 | 3573 | 5655 | 3216 |
| PB | 71261 | 4782 | 8464 | 7248 | 10020 | 10605 | 9205 | 5638 | 4569 | 3435 | 5022 | 2273 |
| PE | 61530 | 2592 | 5355 | 4815 | 6668 | 10162 | 7815 | 5863 | 5548 | 4819 | 5185 | 2708 |
| PI | 9723 | 436 | 616 | 562 | 826 | 1310 | 1652 | 951 | 947 | 1043 | 956 | 424 |
| PR | 263162 | 9187 | 27897 | 34148 | 42341 | 38325 | 29255 | 20708 | 19141 | 20906 | 16075 | 5179 |
| RJ | 40134 | 821 | 1999 | 3205 | 5026 | 4735 | 4496 | 4349 | 4792 | 4332 | 4208 | 2171 |
| RN | 20562 | 950 | 1922 | 2185 | 2272 | 2478 | 2413 | 2128 | 2055 | 1699 | 1743 | 717 |
| RO | 2955 | 38 | 576 | 320 | 460 | 377 | 377 | 392 | 325 | 65 | 20 | 5 |
| RR | 223 | 10 | 40 | 30 | 29 | 25 | 10 | 0 | 9 | 2 | 66 | 2 |
| RS | 170419 | 6929 | 14654 | 18129 | 19147 | 24800 | 23296 | 15747 | 16636 | 14648 | 13023 | 3410 |
| SC | 235713 | 12738 | 21750 | 22869 | 30480 | 29802 | 24740 | 23537 | 24349 | 24279 | 17207 | 3962 |
| SE | 1861 | 93 | 198 | 218 | 286 | 400 | 123 | 91 | 95 | 124 | 169 | 64 |
| SP | 398175 | 8490 | 27140 | 35765 | 51404 | 50606 | 44183 | 39748 | 41603 | 41280 | 41032 | 16924 |
| TO | 20433 | 857 | 1112 | 513 | 1171 | 1489 | 1838 | 1774 | 4455 | 5238 | 1715 | 271 |
