# Supplementary figures and images for: Prevalence and temporal trends in prepregnancy nutritional status and gestational weight gain of adult women followed in the Brazilian Food and Nutrition Surveillance System from 2008 to 2018
Source: Matern Child Nutr. 2021 Jul 13;18(1):e13240. doi: 10.1111/mcn.13240 (PMC8710119; doi:10.1111/mcn.13240)

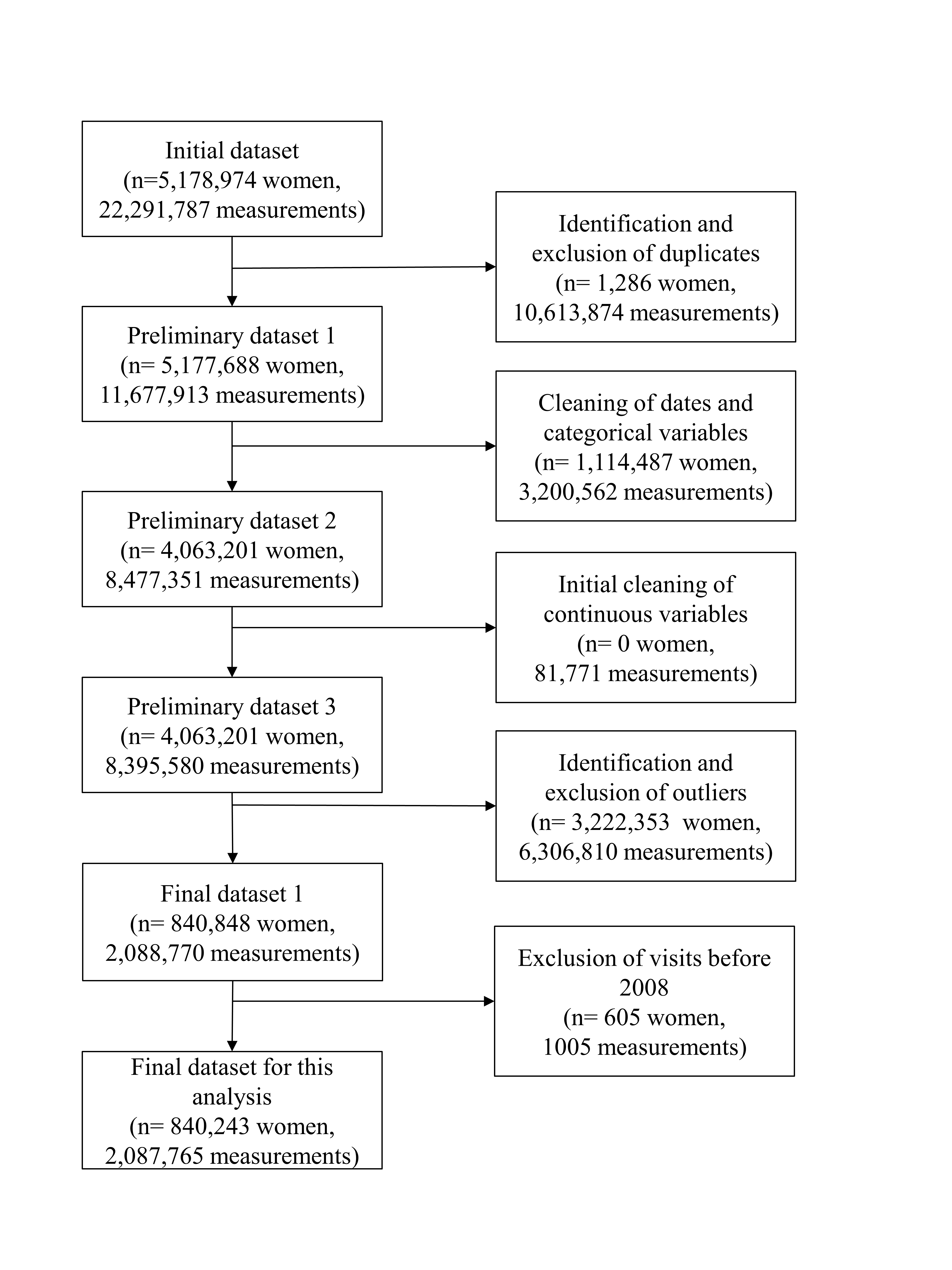

Supplement: Supplementary file 2 — Figure S1. Flowchart for the cleaning steps and constitution of the dataset used in the analyses. [file MCN-18-e13240-s002.tif]

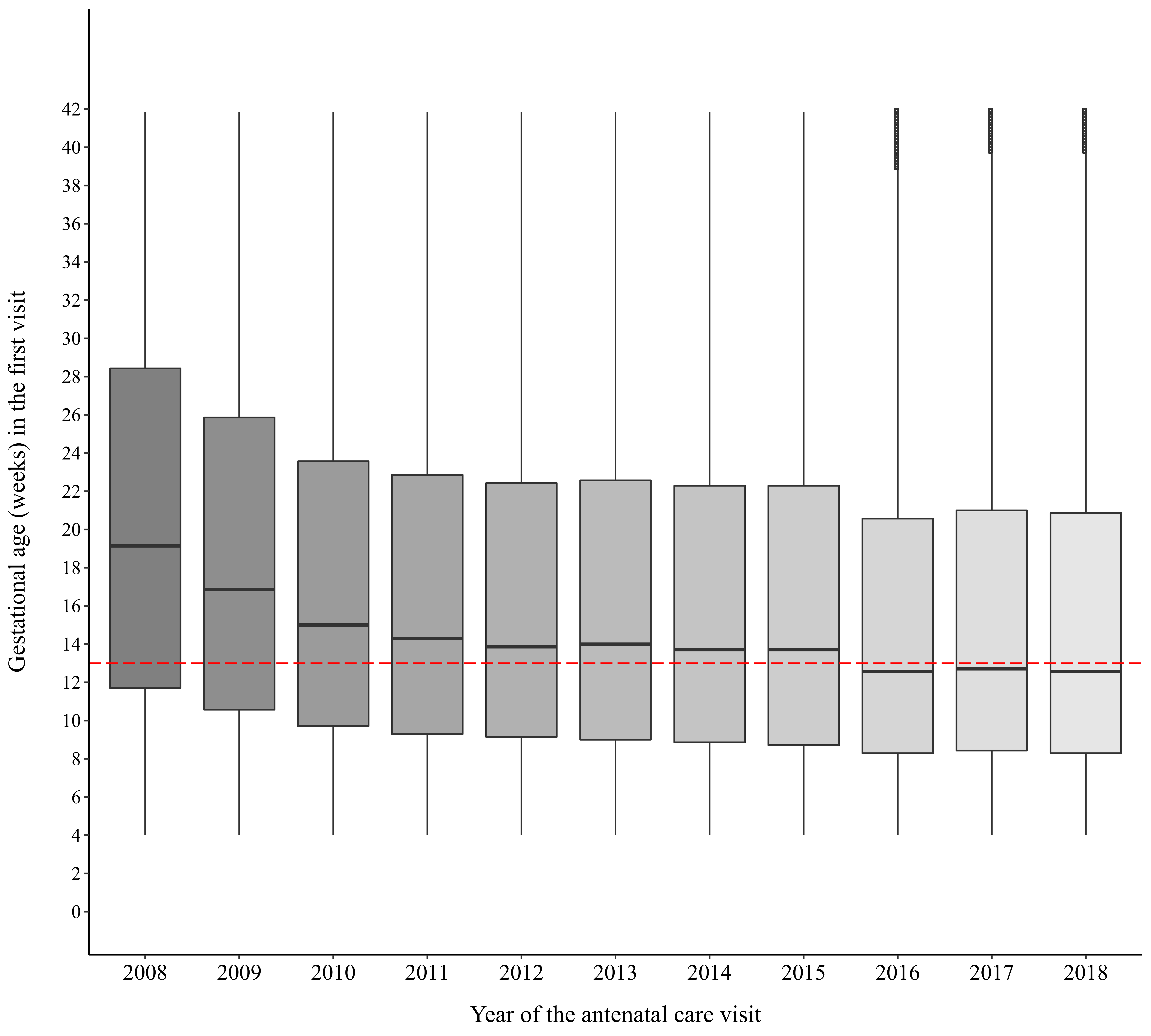

Supplement: Supplementary file 3 — Figure S2. Median and interquartile ranges for gestational age in the first prenatal visit in the Food and Nutrition Surveillance System (SISVAN), 2008–2018. [file MCN-18-e13240-s001.tiff]
